# 4-Carboxy­pyridinium 3-carb­oxy-4-hydroxy­benzene­sulfonate

**DOI:** 10.1107/S1600536808015882

**Published:** 2008-06-07

**Authors:** Zhong-long Wang, Kai-lun Yao, Zu-li Liu, Hui-jin Xu

**Affiliations:** a Department of Physics, Huazhong University of Science and Technology, Wuhan 430074, People’s Republic of China; b Science College, China Three Gorges University, Yichang 443002, People’s Republic of China

## Abstract

Cocrystallization of 4-carboxy­pyridine (4-CPY) and 5-sulfosalicylic acid (5-H_2_SSA) yields the title salt, C_6_H_6_NO_2_^+^·C_7_H_5_O_6_S^−^. In the crystal structure, the components of the salt are linked by a combination of inter­molecular O—H⋯O and N—H⋯O, and weak C—H⋯O hydrogen bonds, forming a three-dimensional framework.

## Related literature

For related literature, see: Aakeröy & Salmon (2005 ▶); Meng *et al.* (2007 ▶, 2008 ▶); Fan *et al.* (2005 ▶); Smith *et al.* (2006 ▶).
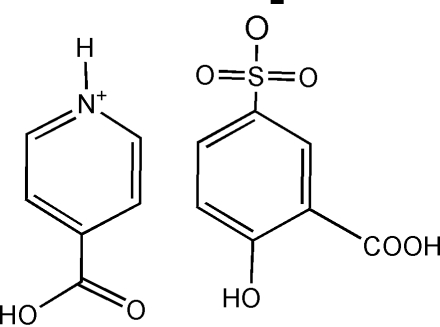


## Experimental

### 

#### Crystal data

C_6_H_6_NO_2_^+^·C_7_H_5_O_6_S^−^
*M* *_r_* = 341.29
Orthorhombic, 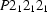

*a* = 6.6358 (6) Å
*b* = 13.0514 (12) Å
*c* = 16.7415 (14) Å
*V* = 1449.9 (2) Å^3^
*Z* = 4
Mo *K*α radiation
μ = 0.27 mm^−1^
*T* = 298 (2) K
0.30 × 0.04 × 0.02 mm

#### Data collection

Bruker SMART APEX CCD area-detector diffractometer
Absorption correction: none
13287 measured reflections
2545 independent reflections
1522 reflections with *I* > 2σ(*I*)
*R*_int_ = 0.143

#### Refinement

*R*[*F* ^2^ > 2σ(*F* ^2^)] = 0.072
*wR*(*F* ^2^) = 0.168
*S* = 0.97
2545 reflections
220 parameters
5 restraints
H atoms treated by a mixture of independent and constrained refinement
Δρ_max_ = 0.45 e Å^−3^
Δρ_min_ = −0.37 e Å^−3^
Absolute structure: Flack (1983 ▶), 1056 Friedel pairs
Flack parameter: −0.1 (2)

### 

Data collection: *SMART* (Bruker, 2001 ▶); cell refinement: *SAINT-Plus* (Bruker, 2001 ▶); data reduction: *SAINT-Plus*; program(s) used to solve structure: *SHELXS97* (Sheldrick, 2008 ▶); program(s) used to refine structure: *SHELXL97* (Sheldrick, 2008 ▶); molecular graphics: *PLATON* (Spek, 2003 ▶); software used to prepare material for publication: *PLATON*.

## Supplementary Material

Crystal structure: contains datablocks global, I. DOI: 10.1107/S1600536808015882/lh2633sup1.cif

Structure factors: contains datablocks I. DOI: 10.1107/S1600536808015882/lh2633Isup2.hkl

Additional supplementary materials:  crystallographic information; 3D view; checkCIF report

## Figures and Tables

**Table 1:** Hydrogen-bond geometry (Å, °)

*D*—H⋯*A*	*D*—H	H⋯*A*	*D*⋯*A*	*D*—H⋯*A*
O1—H1⋯O5^i^	0.82 (2)	1.82 (2)	2.632 (5)	170 (7)
N1—H1*A*⋯O2^ii^	0.85 (2)	2.57 (5)	3.140 (7)	125 (5)
C3—H3⋯O3^ii^	0.93	2.51	3.379 (7)	156
O3—H3*A*⋯O6^iii^	0.82 (2)	2.57 (5)	3.140 (5)	127 (6)
O7—H7⋯O4^iv^	0.83 (5)	1.87 (4)	2.654 (5)	155 (8)
C10—H10⋯O8^v^	0.93	2.46	3.152 (8)	132
C11—H11⋯O4^vi^	0.93	2.57	3.257 (7)	131
N1—H1*A*⋯O6	0.85 (2)	2.14 (3)	2.902 (6)	149 (5)
O3—H3*A*⋯O2	0.82 (2)	1.90 (4)	2.626 (5)	146 (6)
C10—H10⋯O4	0.93	2.54	3.331 (7)	143

Symmetry codes: (i) 
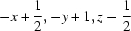
; (ii) 
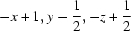
; (iii) 
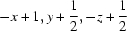
; (iv) 
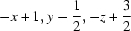
; (v) 
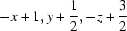
; (vi) 
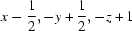
.

**Fig. 1.**
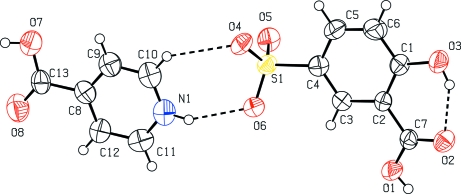
Molecular structure of (I), showing the atom-numbering scheme. Displacement ellipsoids are drawn at the 50% probability level. H-bonds are shown as dashed lines.

**Fig. 2.**
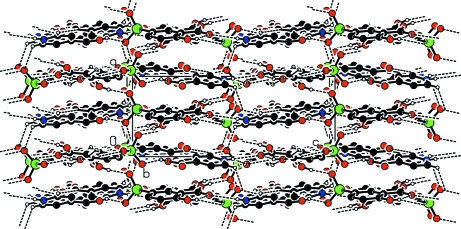
Part of the crystal structure of (I), showing the formation of the three-dimensional framework structure. Hydrogen bonds are shown as dashed lines. For the sake of clarity, H atoms not involved in the motif have been omitted from the drawing.

## supplementary crystallographic information

### Comment

As awareness of the importance of pharmaceutical molecular adducts grows, it becomes imperative to fully understand and investigate the intermolecular interactions in a binary, ternary or multi-component organic adducts (Aakeröy and Salmon, 2005). 5-sulfosalicylic acid (5-H_2_SSA), is a particularly strong acid which is capable of donating its sulfonic protons to many N-containing heterocycles, forming organic salts (Smith *et al.*, 2006; Meng *et al.*, 2007 and 2008; Fan *et al.*, 2005). With the aim of gaining more insight into hydrogen-bonding interactions involving 5-H_2_SSA and pyridine derivatives, we report here the molecular and supramolecular structure of the title compound.

In the asymmetric unit of title compound (I), contains one 5-HSSA^- ^and one 4-CPY^+^ ion. Similar to the analogous organic adducts reported (Meng *et al.*, 2007), the H atom is transferred from the sulfonic acid group to the pyridine N atom forming an 1:1 organic salt.

In the crystal structure, the component ions are linked by a combination of O—H···O, N—H···O and C—H···O hydrogen bonds (Table 1), forming a three-dimensional network (Fig.2). An analysis using *PLATON* (Spek, 2003), showed that there are no other interactions (*e.g.* C—H···π and π-π) observed in the crystal structure.

### Experimental

All reagents and solvents were used as obtained without further purification. Equivalent molar amount of 4-carboxypyridine and 5-sulfosalicylic acid dihydrate were dissolved in 95% methanol (20 ml). The mixture was stirred for 10 minutes at 330 K and then filtered. Colorless needles of (I) suitable for single-crystal X-ray diffraction analysis grew at the bottom of the vessel in one week after slow evaporation of the solution.

### Refinement

Owing to the poor quality the crystal selected for diffraction, conventional least squares refinement of the structural model gave *R*_1 _= 0.072. We attempted to select better crystals for diffraction, but none were an improvement. The title compound is racemic in solution but spontaneously resolved upon crystallization. The absolute configuration of the molecules in the crystal selected was readily determined and the configuration has no chemical significance.

H atoms bonded to C atoms were positioned geometrically with C–H = 0.93 Å (aromatic) and refined in a riding mode [*U*_iso_(H) = 1.2*U*_eq_(aromatic C)]. H atoms bonded to N and O atoms were found in Fourier difference maps and refined with the constraints of N—H = 0.86 (2) Å,*O*—H = 0.82 (2) Å, and *U*_iso_(H) = 1.2*U*_eq_(N) or 1.5*U*_eq_(O)].

### Crystal data

**Table:**

C_6_H_6_NO_2_^+^·C_7_H_5_O_6_S^−^	*F*(000) = 704
*M**_r_* = 341.29	*D*_x_ = 1.563 Mg m^−^^3^
Orthorhombic, *P*2_1_2_1_2_1_	Mo *K*α radiation, λ = 0.71073 Å
Hall symbol: P 2ac 2ab	Cell parameters from 950 reflections
*a* = 6.6358 (6) Å	θ = 3.1–19.7°
*b* = 13.0514 (12) Å	µ = 0.27 mm^−^^1^
*c* = 16.7415 (14) Å	*T* = 298 K
*V* = 1449.9 (2) Å^3^	Needle, colorless
*Z* = 4	0.30 × 0.04 × 0.02 mm

### Data collection

**Table:**

Bruker SMART APEX CCD area-detector diffractometer	1522 reflections with *I* > 2σ(*I*)
Radiation source: fine focus sealed Siemens Mo tube	*R*_int_ = 0.143
graphite	θ_max_ = 25.0°, θ_min_ = 2.9°
0.3° wide ω exposures scans	*h* = −7→7
13287 measured reflections	*k* = −15→15
2545 independent reflections	*l* = −19→18

### Refinement

**Table:**

Refinement on *F*^2^	Secondary atom site location: difference Fourier map
Least-squares matrix: full	Hydrogen site location: inferred from neighbouring sites
*R*[*F*^2^ > 2σ(*F*^2^)] = 0.072	H atoms treated by a mixture of independent and constrained refinement
*wR*(*F*^2^) = 0.168	*w* = 1/[σ^2^(*F*_o_^2^) + (0.0554*P*)^2^] where *P* = (*F*_o_^2^ + 2*F*_c_^2^)/3
*S* = 0.97	(Δ/σ)_max_ < 0.001
2545 reflections	Δρ_max_ = 0.45 e Å^−^^3^
220 parameters	Δρ_min_ = −0.37 e Å^−^^3^
5 restraints	Absolute structure: Flack (1983), 1056 Friedel pairs
Primary atom site location: structure-invariant direct methods	Flack parameter: −0.1 (2)

### Special details

**Table:**

Geometry. All e.s.d.'s (except the e.s.d. in the dihedral angle between two l.s. planes) are estimated using the full covariance matrix. The cell e.s.d.'s are taken into account individually in the estimation of e.s.d.'s in distances, angles and torsion angles; correlations between e.s.d.'s in cell parameters are only used when they are defined by crystal symmetry. An approximate (isotropic) treatment of cell e.s.d.'s is used for estimating e.s.d.'s involving l.s. planes.
Refinement. Refinement of *F*^2^ against ALL reflections. The weighted *R*-factor *wR* and goodness of fit *S* are based on *F*^2^, conventional *R*-factors *R* are based on *F*, with *F* set to zero for negative *F*^2^. The threshold expression of *F*^2^ > σ(*F*^2^) is used only for calculating *R*-factors(gt) *etc*. and is not relevant to the choice of reflections for refinement. *R*-factors based on *F*^2^ are statistically about twice as large as those based on *F*, and *R*- factors based on ALL data will be even larger.

### Fractional atomic coordinates and isotropic or equivalent isotropic displacement parameters (Å^2^)

**Table:**

	*x*	*y*	*z*	*U*_iso_*/*U*_eq_	
C1	0.5027 (9)	0.7437 (4)	0.2884 (4)	0.0404 (15)	
C2	0.4566 (8)	0.6435 (4)	0.2645 (3)	0.0334 (13)	
C3	0.4291 (9)	0.5677 (4)	0.3235 (3)	0.0362 (13)	
H3	0.3988	0.5009	0.3082	0.043*	
C4	0.4464 (9)	0.5908 (4)	0.4022 (3)	0.0410 (15)	
C5	0.4945 (12)	0.6910 (4)	0.4256 (4)	0.0563 (18)	
H5	0.5083	0.7069	0.4794	0.068*	
C6	0.5212 (12)	0.7655 (4)	0.3689 (4)	0.062 (2)	
H6	0.5524	0.8319	0.3848	0.074*	
C7	0.4373 (9)	0.6190 (4)	0.1788 (3)	0.0393 (14)	
O1	0.3740 (8)	0.5263 (3)	0.1634 (2)	0.0556 (12)	
H1	0.335 (11)	0.517 (5)	0.1176 (19)	0.083*	
O2	0.4714 (6)	0.6826 (3)	0.1260 (2)	0.0496 (11)	
O3	0.5284 (8)	0.8216 (3)	0.2371 (2)	0.0554 (13)	
H3A	0.524 (13)	0.799 (5)	0.1914 (18)	0.083*	
O4	0.5754 (7)	0.5082 (3)	0.5343 (2)	0.0524 (11)	
O5	0.2154 (6)	0.5225 (3)	0.5150 (2)	0.0519 (11)	
O6	0.4088 (7)	0.3976 (2)	0.4391 (2)	0.0474 (10)	
S1	0.4079 (2)	0.49700 (10)	0.47738 (8)	0.0424 (4)	
C8	0.4478 (9)	0.1284 (4)	0.6933 (4)	0.0466 (15)	
C9	0.5068 (10)	0.2291 (5)	0.6931 (4)	0.0551 (18)	
H9	0.5412	0.2611	0.7409	0.066*	
C10	0.5153 (10)	0.2820 (5)	0.6234 (4)	0.0538 (17)	
H10	0.5557	0.3503	0.6230	0.065*	
C11	0.4091 (10)	0.1365 (5)	0.5529 (4)	0.0531 (16)	
H11	0.3762	0.1060	0.5045	0.064*	
C12	0.4005 (9)	0.0804 (4)	0.6229 (4)	0.0474 (15)	
H12	0.3635	0.0117	0.6223	0.057*	
C13	0.4301 (10)	0.0658 (5)	0.7697 (4)	0.0491 (16)	
N1	0.4651 (8)	0.2351 (4)	0.5553 (4)	0.0533 (14)	
H1A	0.480 (8)	0.267 (4)	0.511 (2)	0.064*	
O7	0.4546 (8)	0.1208 (3)	0.8342 (3)	0.0669 (14)	
H7	0.478 (13)	0.078 (4)	0.870 (3)	0.100*	
O8	0.3980 (8)	−0.0253 (3)	0.7684 (3)	0.0715 (14)	

### Atomic displacement parameters (Å^2^)

**Table:**

	*U*^11^	*U*^22^	*U*^33^	*U*^12^	*U*^13^	*U*^23^
C1	0.037 (4)	0.045 (3)	0.040 (3)	−0.008 (3)	0.006 (3)	0.005 (3)
C2	0.025 (3)	0.041 (3)	0.035 (3)	0.003 (2)	0.000 (3)	0.004 (2)
C3	0.028 (3)	0.039 (3)	0.042 (4)	−0.002 (2)	0.002 (3)	0.000 (3)
C4	0.034 (4)	0.046 (3)	0.043 (4)	−0.003 (3)	0.007 (3)	0.002 (3)
C5	0.077 (5)	0.054 (3)	0.038 (3)	−0.006 (4)	−0.006 (4)	−0.005 (3)
C6	0.085 (6)	0.043 (3)	0.057 (4)	−0.017 (4)	0.009 (4)	−0.003 (3)
C7	0.035 (4)	0.043 (3)	0.040 (4)	0.009 (3)	0.001 (3)	0.004 (3)
O1	0.079 (3)	0.047 (2)	0.041 (3)	−0.011 (2)	−0.012 (3)	−0.003 (2)
O2	0.048 (3)	0.057 (2)	0.043 (2)	−0.007 (2)	0.002 (2)	0.003 (2)
O3	0.075 (4)	0.050 (2)	0.042 (2)	−0.010 (2)	−0.003 (3)	0.008 (2)
O4	0.054 (3)	0.066 (2)	0.037 (2)	−0.001 (2)	−0.012 (2)	−0.003 (2)
O5	0.040 (3)	0.064 (3)	0.052 (3)	0.002 (2)	0.021 (2)	0.008 (2)
O6	0.054 (3)	0.047 (2)	0.042 (2)	0.000 (2)	0.000 (3)	−0.0028 (18)
S1	0.0461 (9)	0.0454 (8)	0.0356 (8)	−0.0018 (8)	0.0026 (7)	0.0006 (7)
C8	0.028 (4)	0.051 (3)	0.061 (4)	0.008 (3)	−0.002 (3)	0.002 (3)
C9	0.044 (4)	0.059 (4)	0.063 (5)	0.002 (3)	−0.006 (4)	0.003 (3)
C10	0.031 (4)	0.060 (4)	0.070 (5)	0.000 (3)	−0.001 (4)	0.007 (4)
C11	0.030 (4)	0.070 (4)	0.059 (4)	0.001 (3)	0.002 (4)	−0.009 (3)
C12	0.029 (3)	0.060 (4)	0.053 (4)	−0.001 (3)	−0.003 (4)	0.006 (4)
C13	0.039 (4)	0.053 (4)	0.056 (4)	0.010 (3)	−0.006 (4)	0.009 (3)
N1	0.033 (3)	0.065 (4)	0.062 (4)	0.003 (3)	0.008 (3)	0.011 (3)
O7	0.081 (4)	0.064 (3)	0.055 (3)	−0.001 (3)	−0.007 (3)	0.006 (2)
O8	0.077 (4)	0.062 (3)	0.075 (3)	−0.008 (3)	−0.011 (3)	0.015 (2)

### Geometric parameters (Å, °)

**Table:**

C1—O3	1.343 (6)	O5—S1	1.462 (4)
C1—C6	1.382 (8)	O6—S1	1.446 (3)
C1—C2	1.401 (7)	C8—C12	1.370 (8)
C2—C3	1.409 (7)	C8—C9	1.371 (8)
C2—C7	1.477 (7)	C8—C13	1.523 (8)
C3—C4	1.357 (7)	C9—C10	1.359 (8)
C3—H3	0.9300	C9—H9	0.9300
C4—C5	1.401 (7)	C10—N1	1.336 (8)
C4—S1	1.775 (5)	C10—H10	0.9300
C5—C6	1.371 (8)	C11—N1	1.340 (7)
C5—H5	0.9300	C11—C12	1.383 (8)
C6—H6	0.9300	C11—H11	0.9300
C7—O2	1.233 (6)	C12—H12	0.9300
C7—O1	1.306 (6)	C13—O8	1.209 (7)
O1—H1	0.82 (2)	C13—O7	1.306 (7)
O3—H3A	0.82 (2)	N1—H1A	0.85 (2)
O4—S1	1.472 (4)	O7—H7	0.83 (5)
			
O3—C1—C6	117.1 (5)	O6—S1—C4	107.7 (2)
O3—C1—C2	123.5 (5)	O5—S1—C4	105.9 (3)
C6—C1—C2	119.3 (5)	O4—S1—C4	106.4 (3)
C1—C2—C3	118.9 (5)	C12—C8—C9	120.1 (6)
C1—C2—C7	119.9 (5)	C12—C8—C13	117.4 (5)
C3—C2—C7	121.2 (5)	C9—C8—C13	122.5 (6)
C4—C3—C2	120.8 (5)	C10—C9—C8	120.1 (6)
C4—C3—H3	119.6	C10—C9—H9	120.0
C2—C3—H3	119.6	C8—C9—H9	120.0
C3—C4—C5	119.9 (5)	N1—C10—C9	119.3 (6)
C3—C4—S1	121.5 (4)	N1—C10—H10	120.3
C5—C4—S1	118.6 (4)	C9—C10—H10	120.3
C6—C5—C4	119.9 (6)	N1—C11—C12	119.7 (6)
C6—C5—H5	120.1	N1—C11—H11	120.1
C4—C5—H5	120.1	C12—C11—H11	120.1
C5—C6—C1	121.1 (5)	C8—C12—C11	118.5 (5)
C5—C6—H6	119.4	C8—C12—H12	120.8
C1—C6—H6	119.4	C11—C12—H12	120.8
O2—C7—O1	122.8 (5)	O8—C13—O7	125.3 (6)
O2—C7—C2	122.3 (5)	O8—C13—C8	121.7 (6)
O1—C7—C2	114.8 (5)	O7—C13—C8	113.0 (5)
C7—O1—H1	115 (5)	C10—N1—C11	122.3 (6)
C1—O3—H3A	109 (5)	C10—N1—H1A	119 (4)
O6—S1—O5	113.5 (3)	C11—N1—H1A	119 (4)
O6—S1—O4	111.9 (2)	C13—O7—H7	105 (5)
O5—S1—O4	111.0 (2)		

### Hydrogen-bond geometry (Å, °)

**Table:**

*D*—H···*A*	*D*—H	H···*A*	*D*···*A*	*D*—H···*A*
O1—H1···O5^i^	0.82 (2)	1.82 (2)	2.632 (5)	170 (7)
N1—H1A···O2^ii^	0.85 (2)	2.57 (5)	3.140 (7)	125 (5)
C3—H3···O3^ii^	0.93	2.51	3.379 (7)	156.
O3—H3A···O6^iii^	0.82 (2)	2.57 (5)	3.140 (5)	127 (6)
O7—H7···O4^iv^	0.83 (5)	1.87 (4)	2.654 (5)	155 (8)
C10—H10···O8^v^	0.93	2.46	3.152 (8)	132.
C11—H11···O4^vi^	0.93	2.57	3.257 (7)	131.
N1—H1A···O6	0.85 (2)	2.14 (3)	2.902 (6)	149 (5)
O3—H3A···O2	0.82 (2)	1.90 (4)	2.626 (5)	146 (6)
C10—H10···O4	0.93	2.54	3.331 (7)	143.

Symmetry codes: (i) −*x*+1/2, −*y*+1, *z*−1/2; (ii) −*x*+1, *y*−1/2, −*z*+1/2; (iii) −*x*+1, *y*+1/2, −*z*+1/2; (iv) −*x*+1, *y*−1/2, −*z*+3/2; (v) −*x*+1, *y*+1/2, −*z*+3/2; (vi) *x*−1/2, −*y*+1/2, −*z*+1.
